# Radiosensitization effect by HDAC inhibition improves NKG2D-dependent natural killer cytotoxicity in hepatocellular carcinoma

**DOI:** 10.3389/fonc.2022.1009089

**Published:** 2022-09-15

**Authors:** Yu-Fan Liu, Yun Chiang, Feng-Ming Hsu, Chiao-Ling Tsai, Jason Chia-Hsien Cheng

**Affiliations:** ^1^ Division of Radiation Oncology, Department of Oncology, National Taiwan University Hospital, Taipei, Taiwan; ^2^ Graduate Institute of Oncology, College of Medicine, National Taiwan University, Taipei, Taiwan; ^3^ Graduate Institute of Clinical Medicine, College of Medicine, National Taiwan University, Taipei, Taiwan

**Keywords:** hepatocellular carcinoma, radiotherapy, histone deacetylase inhibition, NKG2D ligand, NK cell-mediated cytotoxicity

## Abstract

**Background:**

Hepatocellular carcinoma (HCC) is one of the leading causes of cancer-related death worldwide. Radiotherapy (RT) controls HCC unsatisfactorily and temporarily. Histone deacetylase inhibitor (HDACi) is a heterogeneous group of epigenetic therapeutics with promising anticancer effects and synergism in combination with RT. HDACi modulates natural killer (NK) cell ligand expression on tumor cells, and leads to immune evasion of cancer cells. Expressions of NK group 2D (NKG2D) ligands on cancer cells determine the cytotoxic effect by interacting with NKG2D receptor on NK cells. However, the role of NKG2D signaling in HCC upon combined RT and HDACi remains unclear.

**Method:**

*In vitro* co-culture system with NK cells was tested for human and murine HCC cell lines. Pan-HDACi (panobinostat) and specific HDAC4 knockdown (HDAC4-KD) were used for HDAC inhibition. Clonogenic assay and flow cytometry examined HCC cell survival and NKG2D ligand expression, respectively. Syngeneic mouse model was used to validate the radiosensitizing effect *in vivo*.

**Results:**

Combined RT and HDACi/HDAC4-KD significantly enhanced NK cell-related cytotoxicity and increased NKG2D ligands, MICA/MICB expressions in human and RAE-1/H60 expressions in murine HCC cells. Delayed tumor growth *in vivo* by the combinational treatment of RT and HDACi/HDAC4-KD was shown with the associated NKG2D ligand expressions. However, NKG2D receptor did not significantly change among tumors.

**Conclusion:**

Radiosensitizing effect with combined RT and HDAC inhibition increased the expression of NKG2D ligands in HCC cells and enhanced their susceptibility to NK cell-mediated cytotoxicity. These findings imply the potential use of combined RT/HDACi and NK cell-directed immunotherapy.

## Introduction

Hepatocellular carcinoma (HCC) is one of the leading causes of cancer-related lethality worldwide (1, 2). Radiotherapy (RT) usually controls HCC tumors unsatisfactorily and temporarily, which demands the need of radiosensitizers to improve the therapeutic ratio (3, 4). Conventional RT exerts a direct anti-tumor effect through DNA damage to cancer cells with high-energy particles leading to subsequent cell death (5). With the established effect of immunotherapy on HCC, it is recently studied that RT to treat HCC often causes immune-modifying effects and changes the tumor microenvironment (TME). Thus, radiation may modulate the target expression of immunotherapy (6, 7). Clinical study showed the enhanced RT effect mediated by natural killer (NK) cells after electron irradiation (8). Previous studies also reported the NK group 2D (NKG2D)-dependent antitumor effects of RT against glioblastoma with a significant tumor reduction *in vivo*. Besides, RT could enhance the NK cell receptor, NKG2D expression and NK cell with antitumor cytotoxicity in HCC cells (9, 10).

Histone deacetylase inhibitor (HDACi) is an emerging group of agents which target HDAC and are promising radiosensitizers currently under investigation. Trichostatin A, an HDACi, sensitizes HCC cells to enhanced NK cell-mediated killing effect by regulating immune-related genes, increases NKG2D ligand expressions, and upregulates chemokines responsible for the enhanced infiltration of NK cells into tumor tissues (11). In addition, the use of HDACi was reported to have the immunomodulatory effect on NKG2D system, and was able to reverse the RT-insensitive cancer cells with DNA damage-dependent NKG2D ligands, MICA/MICB^-^, to MICA/MICB^+^ cells as well as sensitize these cells for NK cell mediated cytotoxicity (12, 13). Our previous report demonstrated the potential radiosensitization of HCC with a pan-HDACi (panobinostat) and specific HDAC4 knockdown HCC cells to impair DNA repair process and delay ectopic HCC tumor progression (14).

Radiofrequency ablation (RFA), similar to RT, to neoplastic nodules of liver enhanced the release and exposure of tumor antigens, thereby helped overcome immune tolerance towards cancer cells (15). NK cells expressing higher levels of activated NKG2D receptors and reduced levels of inhibitory NK receptors, together with increased functional activity, e.g. interferon-γ production and cytotoxicity, were found in HCC patients treated with RFA (16). Cancer cells treated with an HDACi upregulated NKG2D ligands in an ATM/ATR-dependent manner, resulted in the increased sensitivity to NK cell lysis, and increased NKG2D ligand levels by the combined RT. HDACi could therefore synergistically enhance the susceptibilities of cancer cells to NK cells (17, 18). However, the role of NKG2D system in HCC upon the combinational use of HDAC inhibition and RT remains unclear. The present study focused on whether radiosensitization with the combined HDAC inhibition and RT can increase the expression of NKG2D ligands in HCC cells and animal model and consequently enhance their susceptibility to NK cell-mediated cytotoxicity.

## Materials and methods

### Cells and materials

The human HCC cell lines PLC5 and Huh7, murine HCC cell lines BNL CL.2 and Hepa 1-6 cells, and human NK-92 cells were purchased from BCRC, Hsinchu, Taiwan. Adherent cell lines were maintained in Dulbecco’s Modified Eagle Medium (DMEM), containing 2 mmol/L L-glutamine and 10% fetal bovine serum. Plasmid encoding short hairpin RNA (shRNA) for HDAC4 (pLKO.1-shHDAC4) and control plasmids for the RNA interference experiments (pLKO.1-shCON) were obtained from the National RNAi Core Facility (Academia Sinica, Taipei, Taiwan). For all experiments described herein, the adherent cells were allowed to attach over a 24-hour period. Subsequently, the experiments were carried out in serum-free medium. Cells were γ-irradiated using cesium-137 source irradiator (CIS-BioInternational, IBA, Saclay, France).

### HCC and NK cell co-culture system

Wild-type or plasmid-transfected HCC cells were placed in six-well plates, with each well containing 1000 cells in 3 mL low-glucose DMEM. After 24 hours, the cells were irradiated, deposited at 37°C, and saturated with humidity of 5% CO_2_ for 4 hours. HCC cells treated with HDACi were washed once by phosphate-buffered saline (PBS) with the replacement of the medium before the subsequent co-culture procedure to remove the toxic effect of HDACi on NK cells. Subsequently, HCC cells were co-cultured with NK cells (0 or 20000 cells/well). Four hours later, the well with co-cultured cells had the DMEM changed to remove the NK cells. Afterwards, the HCC cells were cultured for 7 days at 37°C under saturated humidity of 5% CO_2_ before the subsequent assay.

### Colony-formation assay

Wild-type or plasmid-transfected HCC cells (500-1000/well) were seeded in six-well plates and treated with different doses of radiation (2.5, 5 and 10 Gy) following 24-hour pretreatment with pan-HDAC inhibitor (panobinostat [5 and 10 nM]; provide by Novartis Pharma AG, NIBR, Cambridge, MA, USA) or DMSO vehicle. The cells were cultured for 10 days, fixed, and stained with crystal violet. All colonies (clusters of more than 50 cells) visible to the naked eye were counted. The results were presented as means ± SEM of 3 independent experiments, with duplicate samples for each treatment condition.

### HCC ectopic allograft model

Male, 5- to 6-week-old, BALB/c mice (from BioLASCO Taiwan Co., Ltd, Taipei, Taiwan) were used. In the ectopic allograft HCC tumor model, BNL CL.2 cells (1×10^6^) were injected subcutaneously in the right hind limb of BALB/c mouse. Tumors were allowed to attain an average volume of 100 mm^3^ before RT. The therapeutic effect of RT on this ectopic allograft model was measured by the tumor growth curves. The tumor size was measured in three dimensions twice a week, with volumes calculated using a standard formula: width^2^×length/2. All experimental procedures using these mice were performed in accordance with protocols approved by the National Taiwan University Institutional Animal Care and Use Committee. As the tumors became established, mice were randomized into eight groups to receive the following treatments (1): vehicle (saline solution with 5% DMSO and 1% Tween 80) on days 1-5; (2) panobinostat (25 mg/kg/day of body weight) on days 1-5; (3) vehicle plus 7.5 Gy/day of RT on days 2-4; (4) panobinostat on days 1-5 plus RT on days 2-4; (5) vector-control shRNA (shCON) transfected BNL CL.2 cells; (6) shCON transfected BNL CL.2 cells plus RT on days 2-4; (7) HDAC4 shRNA (shHDAC4) transfected BNL CL.2 cells; (8) shHDAC4 transfected BNL CL.2 cells plus RT on days 2-4. For mice in the RT groups, the tumors were with three 7.5-Gy using a small animal X-ray irradiator (X-RAD SmART [Small Animal RadioTherapy], PRECISION, USA).

### Flow cytometry

The harvested tumor from ectopic allograft model were digested into single cells. Tumoral cells and cultured human or mouse HCC cells (5 × 10^5^) were incubated with fluorescent dye-conjugated mAb (MICA, MICB, RAE1, H60, NKG2D, PD-L1) (R&D Systems, Minneapolis, MN, USA) or the isotype controls at 4°C for 1 h. Then, cells were washed three times with PBS. The fluorescence was finally detected with FACS Calibur (BD Biosciences, San Jose, CA, USA) and analyzed with BD CellQuest Pro software.

### Histological evaluation

Mice from each group were sacrificed on indicated days. The tumor was fixed in 10% neutral buffered formalin and processed for histopathological and immunohistochemical (IHC) staining. After fixation, tumor tissues were embedded in paraffin blocks and sectioned (5 μm). Tumor cells were identified in representative stained sections. Expression of NKG2D (GeneTex, Irvine, CA, USA) was evaluated after IHC staining using specific antibodies according to our previous protocol (14).

### Statistical analysis

Quantitative data were represented as mean ± SD. One-way analysis of variance (ANOVA) with Fisher’s least significant difference method was performed to evaluate the difference between multiple groups. Repeated-measures ANOVA was performed to evaluate the tumor growth curves. All the statistical analyses are performed using GraphPad Prism 8.0 (GraphPad Software). Results with P values of <0.05 were statistically significant.

## Results

### Treatment with HDACi or HDAC4 knockdown radiosensitizes HCC cells and enhances NK cell cytotoxicity

**Figure 1:**
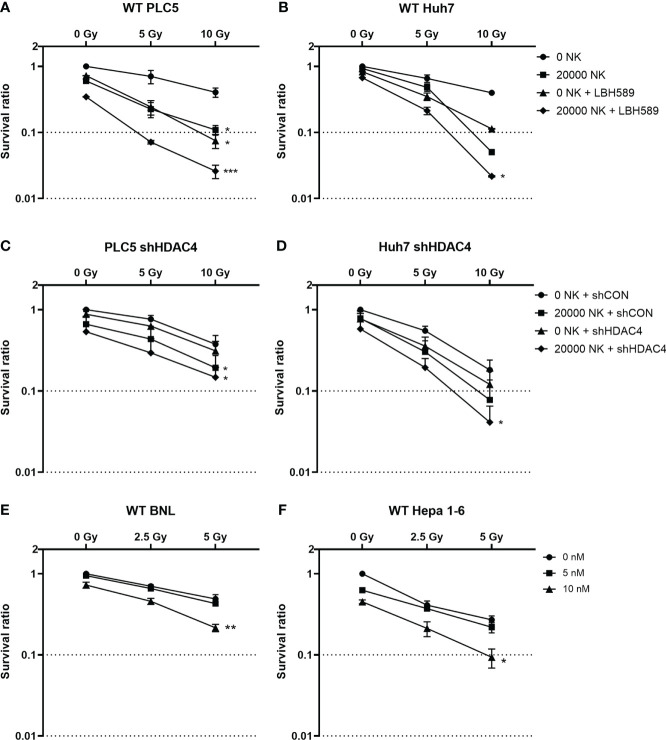
Effect of HDAC inhibition and radiotherapy on clonogenic survival in HCC cells co-cultured with or without natural killer (NK) cells. **(A)** Wild-type PLC5 cells and **(B)** Huh7 cells irradiated with 0, 5 Gy, 10Gy and/or treated with 10 nM of panobinostat (LBH589), a pan-HDAC inhibitor were co-cultured with 0 or 20000 NK cells; **(C)** HDAC4 knockdown (shHDAC4) PLC5 cells and **(D)** Huh7 cells as well as the corresponding shRNA vector-control cells (shCON) irradiated with 0, 5 Gy, 10 Gy were co-cultured with 0 or 20000 NK cells; **(E)** Wild-type BNL cells and **(F)** Hepa 1-6 cells were treated with various doses (0, 5 nM, 10 nM) of HDACi. Significant synergistic effects were observed with combined HDACi or HDAC4 knockdown and radiotherapy on decreased cell survival and enhanced NK cell-related cytotoxicity in HCC cells. Error bars indicate SD, *P < 0.05, **p < 0.01, ***p < 0.001.

To evaluate whether radiosensitization with combined HDAC inhibition and RT can increase the vulnerability of HCC cells and consequently enhance their susceptibility to NK cell-mediated cytotoxicity, the pan-HDAC inhibitor, panobinostat (LBH589) or HDAC4 knockdown combined with various doses of RT were used for the clonogenic survival by co-culture system. Human HCC cell lines (PLC5 and Huh7) were significantly radiosensitized and thus had increased cytotoxicity with cocultured NK cells to HCC under pan-HDACi treatment (**Figures 1A, B**). Similar to HDACi, shHDAC4-transfected human HCC cells had significantly decreased clonogenic survivals when co-cultured with NK cells (**Figures 1C, D**). The combination of pan-HDACi also significantly reduced cell survival in two irradiated murine HCC cell lines (BNL and Hepa 1-6) at 10 nM but not 5 nM (**Figures 1E, F**). The results demonstrated that either HDACi or knockdown of HDAC4 enhanced radiosensitivity in HCC cells and consequently increased NK cell-mediated cytotoxicity with the decreased clonogenic survival in human HCC cells.

### Combined HDACi or HDAC4 knockdown and RT increases the expression of NKG2D ligands and immunogenicity of HCC cells for NK cells

**Figure 2:**
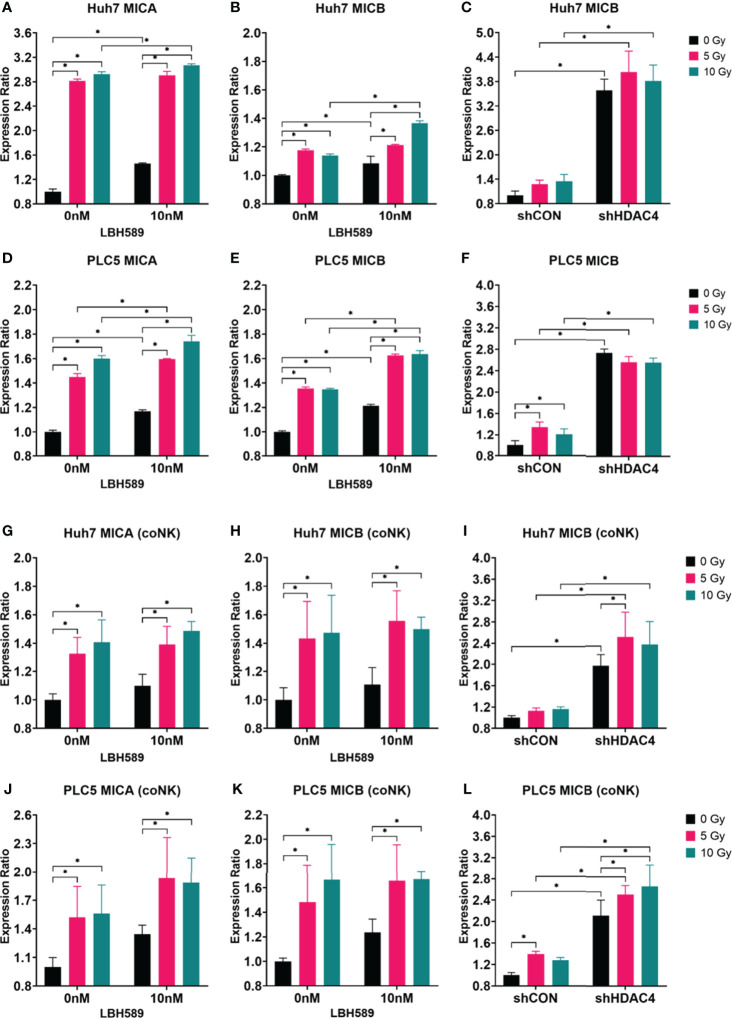
Effect of HDAC inhibition and radiotherapy (RT) on NKG2D ligand expressions in human HCC cells co-cultured with (coNK) or without NK cells. **(A–F)** Wild-type, vector-control shRNA (shCON) or HDAC4 shRNA knockdown (shHDAC4) PLC5 cells and Huh7 cells were treated with 10 nM of a pan-HDAC inhibitor, LBH589; **(G–L)** Wild-type, shCON or shHDAC4 PLC5 cells and Huh7 cells were co-cultured with or without 20000 NK cells. Combined LBH589 or shHDAC4 and RT significantly increased the expressions of NKG2D ligands (MICA/B) of HCC cells with or without the co-cultured NK cells. Error bars indicate SD, *P < 0.05.

To investigate whether radiosensitization with combined HDAC inhibition and RT increases the expressions of NKG2D ligands in HCC cells and consequently enhances their susceptibility to NK cell-mediated cytotoxicity, the pan-HDAC inhibitor (panobinostat) or HDAC4 knockdown combined with various doses of RT were used to induce NKG2D ligands by flow cytometry with MICA/B expressions on PLC5 and Huh7 cells co-cultured with or without NK cells. Our results showed that MICA/B expressions on the surface of HCC cells were significantly increased with HDAC inhibition and/or RT (**Figures 2A** **–F**). The co-culture of HCC cells with NK cells demonstrated the similarly increased expressions of MICA/B (**Figures 2G** **–L**). However, HDAC4 knockdown only increased MICB expression of HCC cells alone or co-cultured with NK cells (**Figures 2C, F, I, L**). Thus, it indicates that the combined HDACi or HDAC4 knockdown and RT significantly increased the immunogenicity of human HCC cells for NK cells.

**Figure 3:**
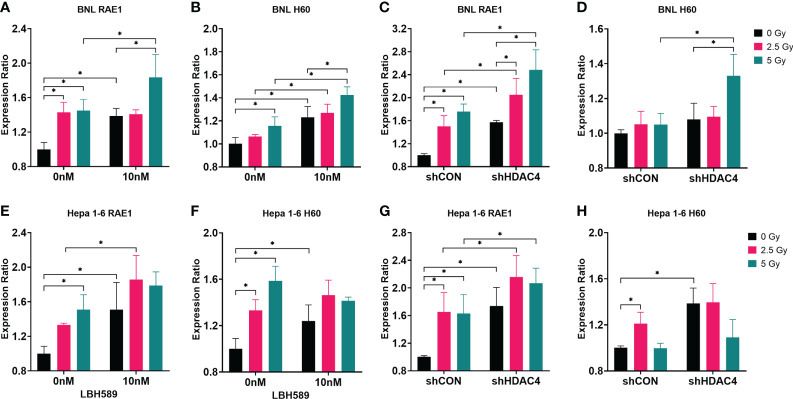
Effect of HDAC inhibition and radiotherapy (RT) on NKG2D ligand expressions in murine HCC cells. **(A, B, E, F)** Wild-type BNL cells and Hepa 1-6 cells were treated with 10 nM of a pan-HDAC inhibitor, LBH589; **(C, D, G, H)** vector-control shRNA (shCON) and HDAC4 shRNA knockdown (shHDAC4) BNL cells and Hepa 1-6 cells were irradiated at 0, 2.5 Gy, or 5 Gy. Combined LBH589 or shHDAC4 and RT significantly increased the expression of NKG2D ligand RAE1 but only partially increased H60 expression in murine HCC cells. Error bars indicate SD, *P < 0.05.

The expressions of murine NKG2D ligands, RAE1 and H60, of BNL and Hepa1-6 cells were analyzed. The results showed that RAE1 expressions in both cell lines were significantly increased with the combined HDACi or HDAC4 knockdown and RT (**Figures 3A, C, E, G**). In contrast, H60 expressions were shown with inconsistent variations between these two cell lines (**Figures 3B, D, F, H**). Hence, the results demonstrated that HDAC inhibition radiosensitizes murine HCC cells and increased the specific immunogenicity (RAE1) for NK cells.

### Treatment with HDACi or HDAC4 knockdown enhances *in vivo* radiosensitivity of ectopic HCC allograft model

**Figure 4:**
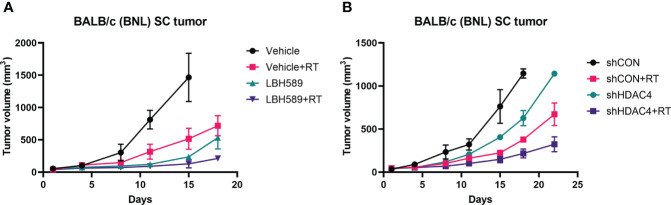
Effect of HDAC inhibition and radiotherapy (RT) on murine allograft tumor control using BNL cells. **(A)** Subcutaneous (SC) allografts were treated with RT and/or a pan-HDAC inhibitor, LBH589. **(B)** Allografts with vector-control shRNA (shCON) and HDAC4 shRNA knockdown (shHDAC4) BNL cells were treated with or without RT. Combination of HDAC inhibition and RT more effectively delayed the tumor growth than either alone.

Next, we tested the therapeutic effects in mice bearing BNL ectopic allografts. Mice bearing subcutaneous wild-type BNL tumors or HDAC4 knockdown tumors were randomized for the indicated treatments. Combined HDACi and RT suppressed the growth of wild-type BNL tumors to a significantly greater extent than RT alone or HDACi alone (**Figure 4A**). RT also significantly delayed the growth of HDAC4 knockdown allografts compared to shCON allografts (**Figure 4B**).

### Combined HDACi but not HDAC4 knockdown and RT increases the surface expressions of specific NKG2D ligands *in vivo*

**Figure 5:**
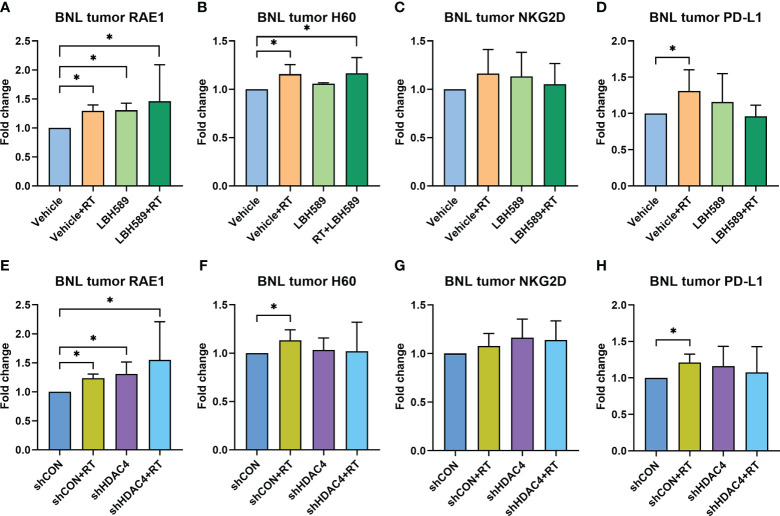
Effect of HDAC inhibitor LBH589 and/or radiotherapy (RT) on tumoral surface expressions of NKG2D ligands **(A)** RAE1, **(B)** H60, **(C)** NKG2D receptor, and **(D)** immune checkpoint molecule PD-L1 in ectopic HCC allografts using wild-type BNL cells, as well as **(E)** RAE1, **(F)** H60, **(G)** NKG2D receptor, and **(H)** immune checkpoint molecule PD-L1 in allografts using HDAC4 shRNA knockdown (shHDAC4) BNL cells. The data represented the fold change compared with vehicle or vector-control shRNA (shCON) group. Error bars indicate SD, *P < 0.05.

**Figure 6:**
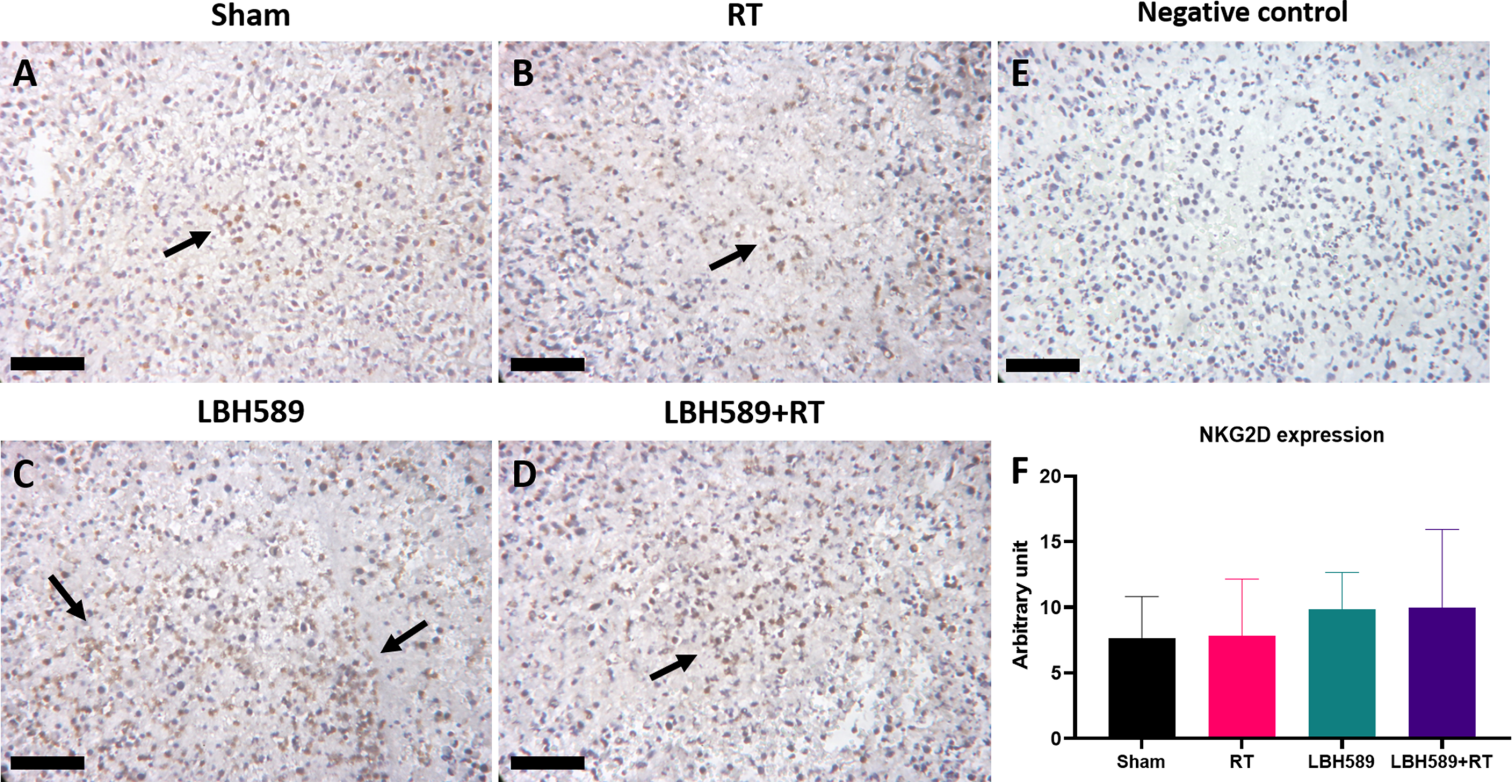
Immunohistochemistry (IHC) staining of NKG2D expression in tumor tissues of **(A)** sham, **(B)** radiotherapy (RT), **(C)** pan-HDAC inhibitor (LBH589), **(D)** combined treatment groups, as well as **(E)** negative control and **(F)** quantification data. The black arrows indicate positive cells. Black bar = 25 μm. Error bars indicate SD.

We then examined whether combined HDAC inhibition and RT increases the expressions of NKG2D ligands of tumor in mice bearing BNL ectopic allografts. The expressions of murine NKG2D ligands, RAE1 and H60, of BNL tumors were analyzed by flow cytometry. Compared with sham group, RAE1 expression was significantly increased in RT, HDACi, and combined HDACi and RT groups (**Figure 5A**). H60 expression was significantly increased only in RT and combined HDACi/RT groups (**Figure 5B**). Besides, RT increased the expression of immune checkpoint molecule, PD-L1, while RT combined with HDACi offset this increment (**Figure 5D**). However, there was no significant increase in the NKG2D receptor among groups (**Figures 5C, G**). Notably, RAE1 expression was increased in HDAC4 knockdown tumors with or without RT (**Figure 5E**), but H60 and PD-L1 expressions only responded to RT (**Figures 5F, H**). IHC staining was performed to confirm tumor tissue NKG2D expression and localization, the data were consistent with the flow cytometry analysis, which showed no significant changes among groups (**Figure 6**).

## Discussion

With recent advancement of surgical techniques, RT technology, targeted therapy and immunotherapy, patients with HCC have more treatment options with the significantly improved prognosis. As the importance of external RT gradually increases in multidisciplinary treatment of HCC patients HCC, RT combined with other treatment modalities may provide the utmost benefit of advanced disease (19).

Our current results suggest that radiosensitization with the combined HDAC inhibition and RT can increase the expression of NKG2D ligands both *in vitro* and *in vivo* in our HCC models and consequently enhance the susceptibility of tumor cells to NK cell-mediated cytotoxicity. NK cells play a specific role in the control of tumor growth and metastasis, and provides innate immunity against infection with certain viruses. Activation of NK cells through NKG2D receptor leads to the release of cytokines and chemokines that is capable of inducing inflammatory responses, modulates monocyte, dendritic cells, and granulocyte growth and differentiation, as well as influence subsequent adaptive immune responses (20). In addition, the expression of NKG2D ligands could be induced by proliferative, tumor-suppressor, and stress signaling pathways linked to the tumorigenic processes, pathogenic insults, and treatments by chemotherapy or radiation (21). Surface expression of NKG2D ligands sensitizes tumor cells to immune cell-mediated destruction by engaging NKG2D to activate NK cells and co-stimulate effector T cells initiating an immune response against the incipient tumor (22). NK cell dysfunction is related to the impaired antitumor immune response in HCC (23). Previous studies revealed that down-regulation and/or loss of NKG2D ligands contribute to the resistance of NK cell-mediated eradication of HCC tumor (24, 25).

HDAC inhibitors have been reported to upregulate expression of NKG2D ligands, and enhance NK cell-mediated killing by regulating immune-related genes and chemokines responsible for the increased infiltration of NK cells into tumor tissues (11, 26, 27). RT increases the expression of NKG2D ligands in tumors, and regulates immune cells including NK cells by inducing the secretion of IFN-γ, TNF-α, perforin, and granzyme B of NK cells through the p38-MAPK, ATM, and NF-κB pathways (28). Our previous study demonstrated the inhibition of HDAC4 either genetically by shRNA or pharmaceutically by an HDACi (panobinostat) combined with RT to reduce proliferation of HCC cells and growth of ectopic xenografts *via* specific DNA repair pathway (14). In the current study, we also use the similar strategy to examine the possibility of NK-mediated cytotoxicity in HCC cells and retardation of immunocompetent ectopic allografts. Our data revealed that HDAC inhibition and/or RT increased human NKG2D ligands (MICA/B) and murine NKG2D ligands (RAE1, H60) and decreased clonogenic survivals especially when HCC cells were co-cultured with NK cells. Notably, pan-HDAC inhibitor, LBH589, showed the better effects than specific HDAC4 knockdown (**Figures 1**–**3**). Murine HCC cells were also tested *in vitro*. NKG2D ligands in BNL cells responded significantly to HDACi/RT treatment, while Hepa 1-6 cells had less significant responses (**Figure 3**).

Our *in vivo* results demonstrated the significant delay in tumor growth with combined HDAC inhibition and RT, which was consistent with the *in vitro* data. Similarly pan-HDAC inhibitor showed better suppressive effect than specific HDAC4 knockdown strategy (**Figure 4**). The different aberrant subtypes of HDAC have been proposed for the progression of HCC, and most of the HDACs, including HDAC4, were upregulated in HCC (29). It is reasonable that pan-HDAC inhibitor, panobinostat, in current study showed greater anti-tumor effect than single HDAC4 knockdown in HCC cells/allografts. Some HDACs may hold the effects on both oncogene and tumor suppressor in cancers (e.g. HDAC6 is involved in tumor suppression by non-epigenetic regulation in HCC) (29, 30). The role of specific HDACs in radiosensitization based on immune-modulation still requires further investigation. NKG2D and NKG2D ligand expressions in murine ectopic tumors also showed comparable results to *in vitro* data, with the increased expression of RAE1 either in RT or HDAC inhibition and to a greater extend when combined HDAC inhibition with RT (**Figures 5A, E**). In contrast, H60 only responded to RT or pan-HDACi/RT combination. Specific HDAC4 knockdown did not induce tumoral H60 expression (**Figures 5B, F**). Previous study revealed that RAE1 was highly expressed in BALB/c-oriented cells (e.g. BNL tumors) and was the major NKG2D ligand compared to H60 (31). Again, pan-HDACi showed greater effect on *in vivo* NKG2D ligand expressions than specific HDAC4 knockdown. In addition, using of paraffin sections from SCID mice ectopic tumor model and IHC analysis confirmed that human NKG2D ligands, MICA/B expression also increased after either HDACi/RT alone or combined treatment in the *in vivo* HCC model (**Supplementary Figure 1**).

In this study, NKG2D receptor was not significantly changed among experimental groups (**Figures 5C** **, G**; **Figure 6**). Generally, in NK cells, NKG2D serves as an activating receptor, which itself is able to trigger cytotoxicity. Some CD8+ T cells also express NKG2D, and send co-stimulatory signals to activate them (32). Thus, the data of NKG2D may represent not only the activated NK cells but also some NKG2D positive infiltrating CD8+ T cells. Nevertheless, NKG2D has been reported to promote HCC tumor growth in diethylnitrosamine induced HCC model (33), suggesting the determined NK cell-mediated anti-tumor effects *via* upregulated NKG2D ligands in our study. Of note, the immune checkpoint molecule, PD-L1, was increased in RT group, while RT combined with HDACi offset this increment. PD-L1 -is able to inhibit cytotoxic CD8+ T cells function by interacting with its immune checkpoint protein, PD-1 on T cells. On the other hand, recent research demonstrated that NK cells are potential responders to PD-1/PD-L1 checkpoint blockade and thus affect T cell function (34). It remains unclear that the time sequence of activating these immune cells after RT. Although our data supports RT with the immunosuppressive responses such as upregulated expression of PD-L1 by tumor cells (6), the combined HDAC inhibition may reverse the immunosuppressive effect of RT by revitalizing NK cells and/or cytotoxic T cells to maximize the therapeutic effect.

This study has a few limitations. First, the *in vivo* results of the present study were based on ectopic allograft HCC model, which might not represent the situation of orthotopic HCC model or human HCC. Practical establishment of liver tumor using hydrodynamic tail-vein injection of HCC cells (35) and transgenic mouse that expresses human NKG2D ligands (36) could be used for the future immuno-oncological assessments. Second, the time point for ectopic tumor sampling in our study might not be the perfect one to detect the most differentiating significance in activating NKG2D ligand-receptor axis. The pre-determined tumor size may be selected according to previous report (37) to analyze the most reactive tumor and immune microenvironment in the forthcoming study. Third, RT intensity used in this study was based on our previous work (14); however, recent report suggested that low-dose fractionated RT may be more effective in activating tumor immune microenvironment (38). The lower doses of RT may be tested for further investigation of our model.

In conclusion, our study explored the immunotherapeutic association of HDAC inhibition and NKG2D system activation with radiosensitization of HCC, mainly with MICA/B and RAE1. Combined HDAC inhibition and RT may be a promising basis for modulating tumor immune microenvironment for the potential use of NK cell-directed immunotherapy to augment the therapeutic effect on HCC.

## Data availability statement

The original contributions presented in the study are included in the article/**Supplementary Material**. Further inquiries can be directed to the corresponding authors.

## Ethics statement

The animal study was reviewed and approved by Institutional Animal Care and Use Committee of the National Taiwan University.

## Author contributions

Y-FL, C-LT, and J-HC conceived and designed the experiments. Y-FL, YC, F-MH, and C-LT collected the data. Y-FL, YC, F-MH, C-LT, and J-HC analyzed and interpreted the data. Y-FL and J-HC drafted the manuscript and revised it critically for important intellectual content. All authors contributed to the article and approved the final version of the manuscript.

## Funding

This work was supported in part by grants from National Taiwan University Hospital (NTUH 109-S4758, 110-S4877, 111-S0150), the National Science and Technology Council, Taiwan (MOST 109-2314-B-002 -100 -MY3; MOST 110-2811-B-002-503; MOST 110-2811-B-002-587).

## Acknowledgments

We thank the staff of the First Core Lab and the Eighth Core Lab, Department of Medical Research, National Taiwan University Hospital for technical assistance during the study.

## Conflict of interest

The authors declare that the research was conducted in the absence of any commercial or financial relationships that could be construed as a potential conflict of interest.

## Publisher’s note

All claims expressed in this article are solely those of the authors and do not necessarily represent those of their affiliated organizations, or those of the publisher, the editors and the reviewers. Any product that may be evaluated in this article, or claim that may be made by its manufacturer, is not guaranteed or endorsed by the publisher.
