# Supplementary material for: Radiosensitization effect by HDAC inhibition improves NKG2D-dependent natural killer cytotoxicity in hepatocellular carcinoma
Source: Front Oncol. 2022 Sep 15;12:1009089. doi: 10.3389/fonc.2022.1009089 (PMC9520006; doi:10.3389/fonc.2022.1009089)
Supplement: Supplementary file 1 [file DataSheet_1.docx]

Supplementary Material

***In vivo* ectopic tumor model and immunohistochemistry (IHC) staining analysis**

The paraffin sections of ectopic thigh tumor were used for IHC staining of MICA/B (Abcam, Boston, MA, USA). Briefly, male nonobese diabetic/severe combined immunodeficiency (SCID) mice were injected with Huh7 HCC cells (1×10^6^) subcutaneously into right hind limb. After tumor was established, the mice were divided into groups to receive HDAC inhibitor (LBH589) and/or radiotherapy (RT).

## Supplementary Figures


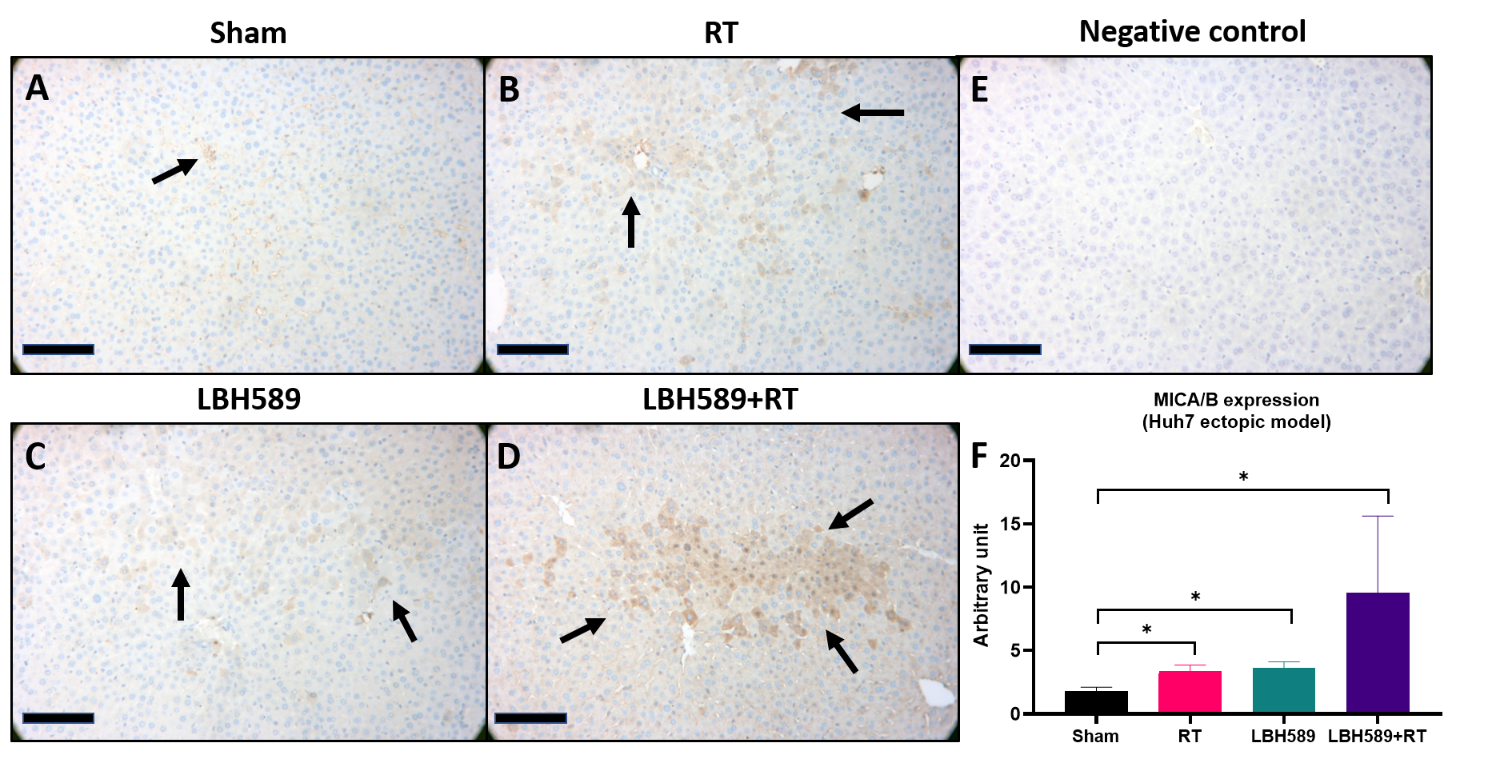


**Supplementary Figure 1.** Immunohistochemistry (IHC) staining of MICA/B expression in tumor tissues of SCID mouse (A) sham, (B) radiotherapy (RT), (C) pan-HDAC inhibitor (LBH589), (D) combined RT and LBH589 groups, as well as (E) negative control and (F) quantification data. The black arrows indicate positive cells. Black bar = 100 μm. Error bars indicate SD. **P*<0.05.
